# Time trends and age-period-cohort analyses on incidence rates of thyroid cancer in Shanghai and Hong Kong

**DOI:** 10.1186/1471-2407-14-975

**Published:** 2014-12-18

**Authors:** Shao-Hua Xie, Juan Chen, Bo Zhang, Feng Wang, Shan-Shan Li, Chang-Hui Xie, Lap-Ah Tse, Jin-Quan Cheng

**Affiliations:** Shenzhen Center for Disease Prevention and Control, Shenzhen, Guangdong Province China; Shenzhen Prevention and Treatment Center for Occupational Diseases, Shenzhen, Guangdong Province China; The Jockey Club School of Public Health and Primary Care, Faculty of Medicine, The Chinese University of Hong Kong, Hong Kong, SAR China; School of Public Health, Sun Yat-sen University, Guangzhou, Guangdong Province China

**Keywords:** Thyroid cancer, Incidence, Time trend, Age–period–cohort analysis, Etiology

## Abstract

**Background:**

Increasing incidence rates of thyroid cancer have been noted worldwide, while the underlying reasons remain unclear.

**Methods:**

Using data from population-based cancer registries, we examined the time trends of thyroid cancer incidence in two largest cities in China, Shanghai and Hong Kong, during the periods 1973–2009 and 1983–2011, respectively. We further performed age-period-cohort analyses to address the possible underlying reasons for the observed temporal trends.

**Results:**

We observed continuous increases in the incidence rates of thyroid cancer in Shanghai and Hong Kong, since the 1980s, in addition to higher incidence rates in the 1970s in both sexes in Shanghai. The age-standardized incidence rate of thyroid cancer increased by 3.1% [95% confidence interval (CI): 1.0%, 5.1%] and 3.8% (95% CI: 1.9%, 5.7%) per year on average, respectively, in Shanghai men and women during the period 1973–2009, while it increased by 2.2% (95% CI: 1.5%, 2.8%) and 2.7% (1.6%, 3.8%) per year on average, respectively, in Hong Kong men and women during the period 1983–2011. We observed global changes in trends across all age groups in similar ways, in addition to varied trends across different generations (birth cohorts).

**Conclusions:**

The increased incidence rates of thyroid cancer in these two Chinese populations during recent decades may be contributable to a combination of the introduction of more sensitive diagnostic techniques and the increasing prevalence of environmental exposures in the populations.

## Background

Thyroid cancer is a malignancy that arises from follicular or parafollicular thyroid cells. A steady increase in the incidence rate of thyroid cancer has been noted in recent decades all over the world, especially in women [1–5]. Previous studies also reported a noteworthy increase in the incidence rate of thyroid cancer in Chinese populations [6–8]. A upward trend was suggested in Hong Kong women, jumping from the 12th in 1999 to 5th most common cancer in 2011 [9]. The increasing incidence rates were indicated by the annual percentage change (APC) of 14.4% and 19.9% in men and women, respectively, in Shanghai since the early 2000s [6].

One possible explanation for the observed increase in the incidence rate of thyroid cancer may be the more frequent use of sensitive diagnostic procedures, such as ultrasound, Doppler examination, CT and MRI scanning, and biochemical markers, during recent decades, which may have increased the detection of thyroid cancers [10, 11]. On the hand, it is also possible attributable to the increasing prevalence of environmental risk factors for thyroid cancer in the population, which was supported by the fact that the increase of thyroid cancer incidence was not restricted to small tumors only [1, 12]. Overall, the underlying causes for the worldwide increase in thyroid cancer incidence remain unclear and are most likely to be multifactorial.

Age-period-cohort analysis is a helpful tool for interpretation of observed temporal trends in disease rates over time, which attempts to sort out the effects of age, calendar period, and birth cohort on disease rates by fitting statistical regression models. Changes in observed disease rate over time in vital registries may be affected by artifacts that alter the estimates of disease rates, such as changes in definition of diseases, improved completeness and quality of reporting systems, and utilization of more frequent and sensitive diagnostic techniques, which may not necessarily indicate true underlying disease burden. In such cases, we would observe a global change in trends that affects the rates across all age groups in a similar way, which is referred to as “period effect”. Temporal changes in disease rates associated with environmental and biological risk factors usually vary across different generations (birth cohorts) being exposed to different exposures, and are known as “cohort effect” [13, 14].

To better understand the temporal trends in the incidence rates of thyroid cancer and the underlying reasons, we examined the time trends of thyroid cancer incidence in two largest cities in China, Shanghai and Hong Kong, during the periods 1973–2009 and 1983–2011, respectively. We further performed age-period-cohort analyses to address the effects of birth cohort and calendar period on the observed temporal trends using a newly developed statistical web tool [15]. Etiological implications were also considered with reference to possible environmental risk factors.

## Methods

We obtained the age- and sex- specific data on all newly diagnosed thyroid cancer cases in Shanghai during 1973–2009 and in Hong Kong during 1983–2011 from Shanghai Cancer Registry and Hong Kong Cancer Registry, respectively. Cancer incidence data from these two population-based cancer registries have been included in the Cancer Incidence in Five Continents series since the 1970s. Age- and sex-specific population data were from Shanghai Municipal Public Security Bureau and the Hong Kong government Census and Statistics Department, respectively.

Age-standardized annual incidence rates were calculated by the direct method using the WHO World Standard Population (1966) as the reference. We used Joinpoint Regression Program developed by US National Cancer Institute (NCI) which identified changing points of the trend and estimate the Annual Percentage Change (APC), under the assumption that the rate changed at a constant percentage every year change linearly on a log scale, for each time segment. The Average Annual Percent Change (AAPC), a weighted average of APCs from the joinpint models with weights equal to the length of the APC interval, was also computed as a summary measure of the trend over the whole observation period [16].

We further performed age-period-cohort regression for each gender to examine the age, period and cohort effects the on incidence rates of thyroid cancer using a web tool for age-period-cohort analysis, which was newly developed by the US NCI [15]. This online web tool can provide the longitudinal age curve in the disease rates which is a smoothing summary curve from observed cohort-specific age-specific rates and considered as superior to age-specific rates derived from cross-sectional data for understanding the age pattern of disease risk. The APCs for each age group, called “local drifts”, can be generated from log-linear regressions. The web tool can also calculate the relative rate in any given calendar period (or birth cohort) versus a referent period (or birth cohort), adjusted for age and non-linear cohort (or period) effects. The central age group, period, and birth cohort were defined as the reference, respectively, in all age-period-cohort analyses. In case of an even number of categories, the reference value was set as the lower of the two central values. Since we only have access to incidence data by 5-year age groups, were used the same 5-year intervals for calendar periods and birth cohorts, so the age-period-cohort analyses were only performed in periods of multiplies of 5, during 1973–2007 in Shanghai and during 1987–2011 in Hong Kong, respectively.

This study was performed with the approval from the Ethics Committee of Shenzhen Center for Disease Prevention and Control and in compliance with the Helsinki Declaration. No information to identify individual subjects was included in the study.

## Results

**Figure 1:**
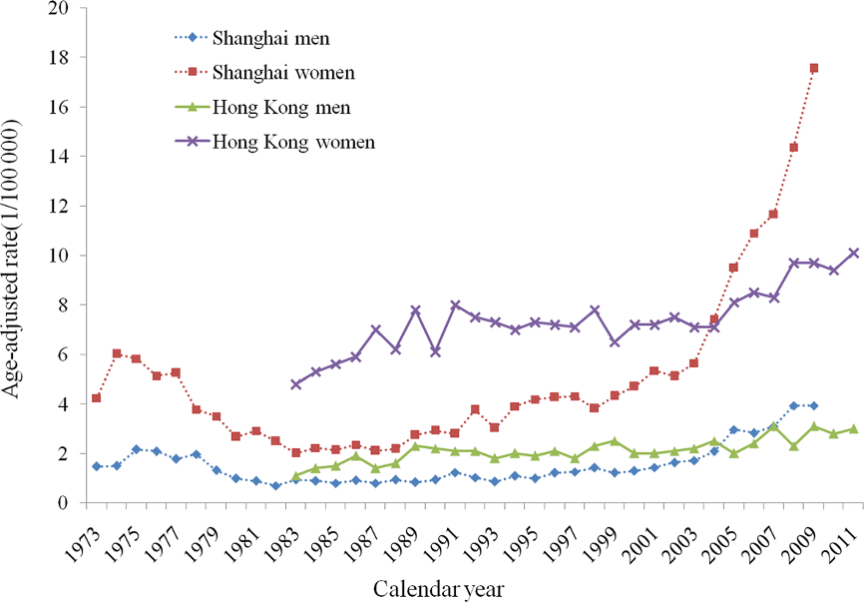
**Time trends of thyroid cancer incidence rates.** Age-standardized incidence rates of thyroid cancer by sex in Shanghai during 1973–2009 and in Hong Kong during 1983–2011, respectively, using WHO World Standard Population 1966 as the reference.

**Figure 2:**
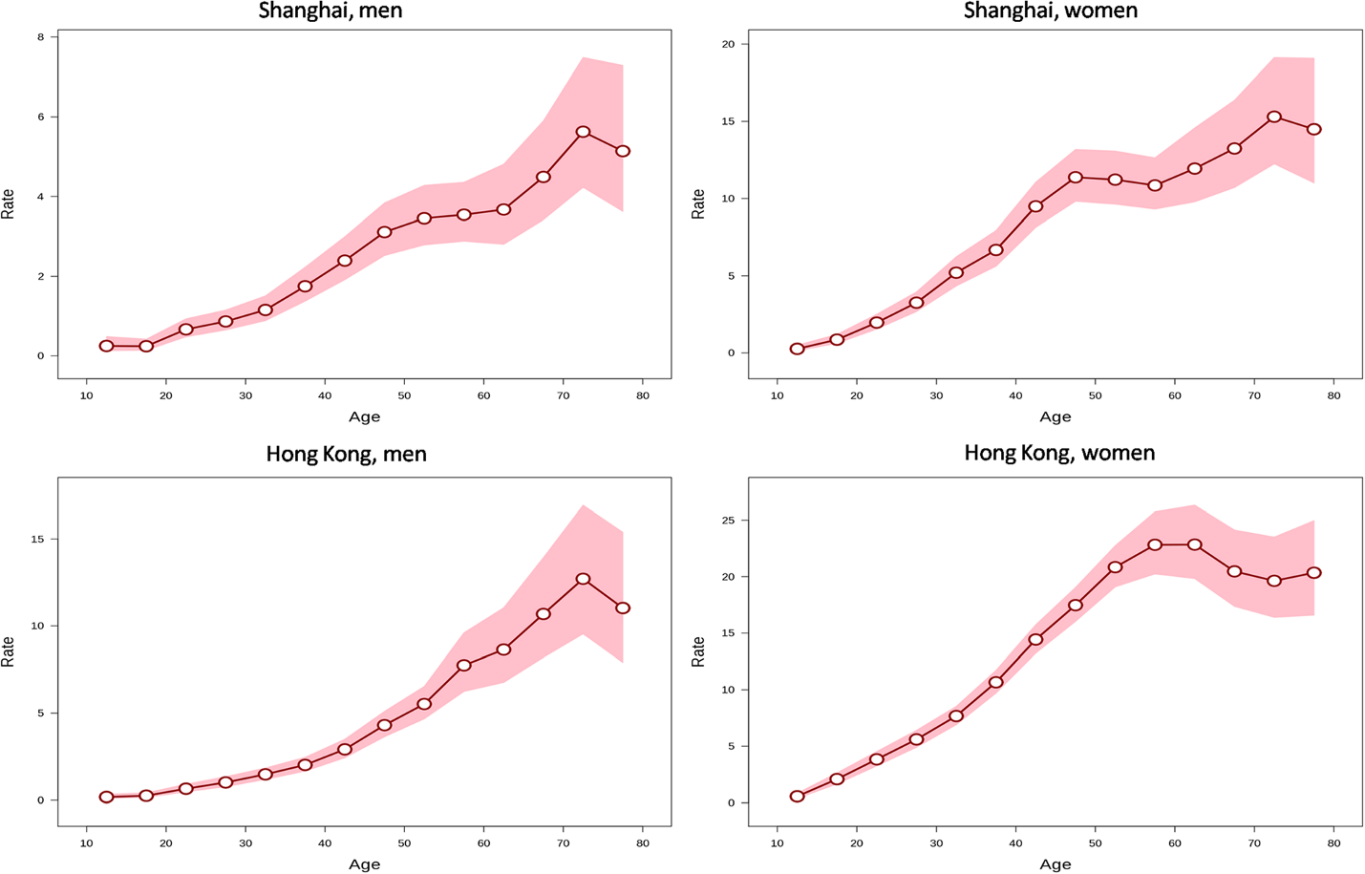
**Longitudinal age curves of thyroid cancer incidence rates.** Longitudinal age curves of the incidence rates (1/100 000) of thyroid cancer and the corresponding 95% confidence intervals by sex in Shanghai and Hong Kong.

**Figure 3:**
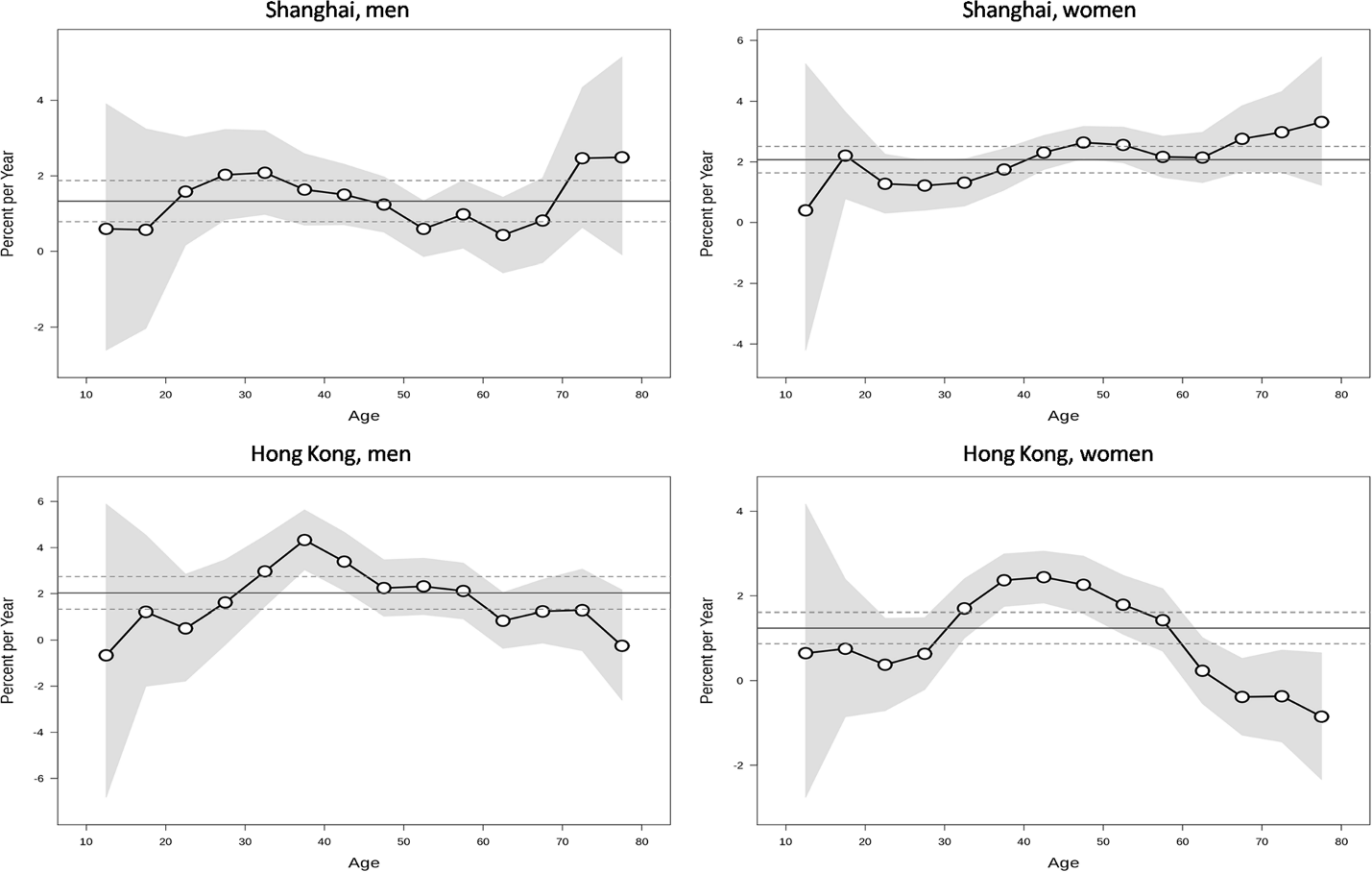
**Local drift values for thyroid cancer incidence rates.** Age group specific annual percent change (%) in the incidence rates of thyroid cancer and the corresponding 95% confidence intervals by sex in Shanghai and Hong Kong.

**Figure 4:**
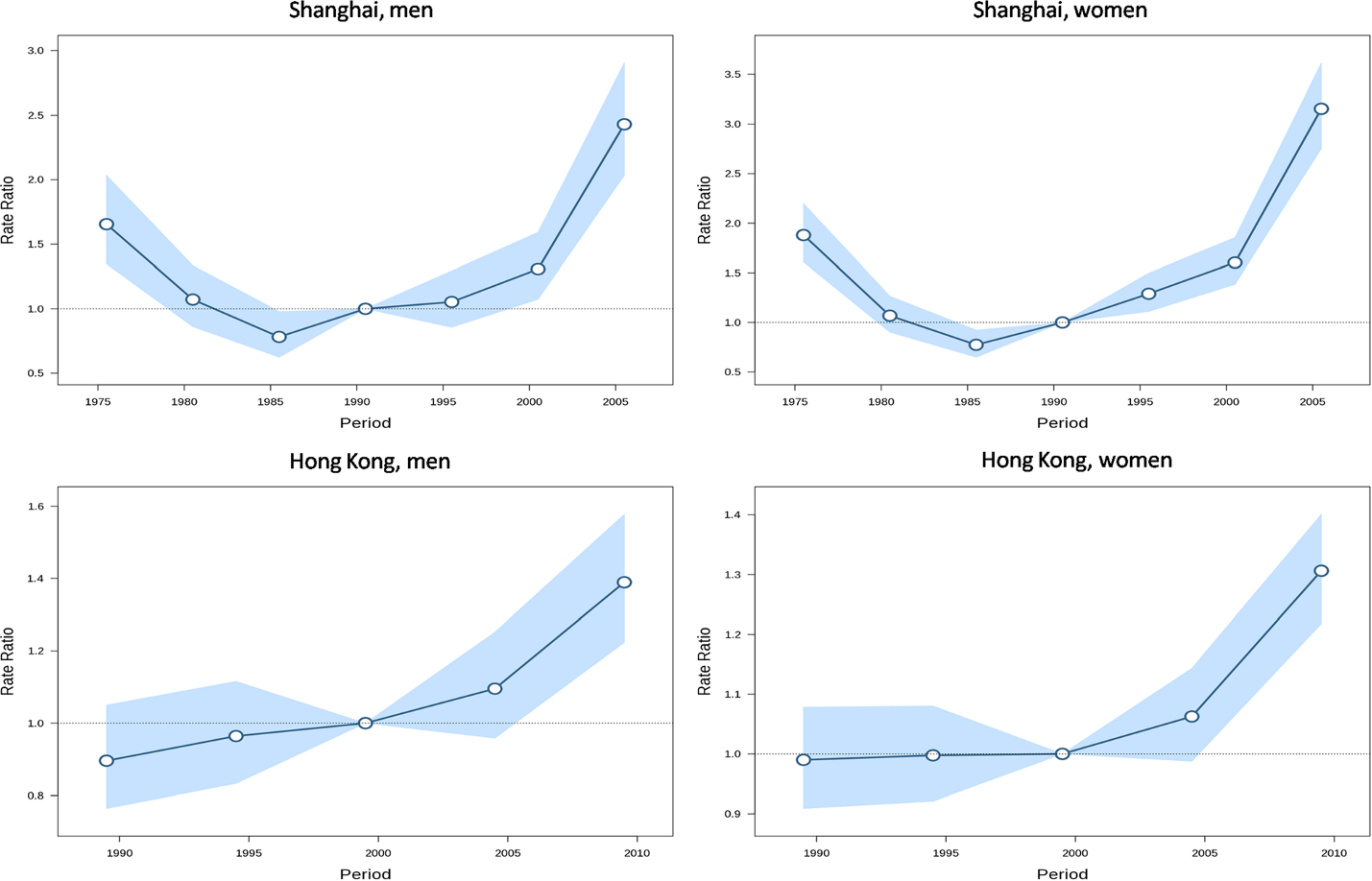
**Period effects on thyroid cancer incidence rates.** Period effects obtained from age-period-cohort analyses for the incidence rates of thyroid cancer and the corresponding 95% confidence intervals by sex in Shanghai and Hong Kong.

**Figure 5:**
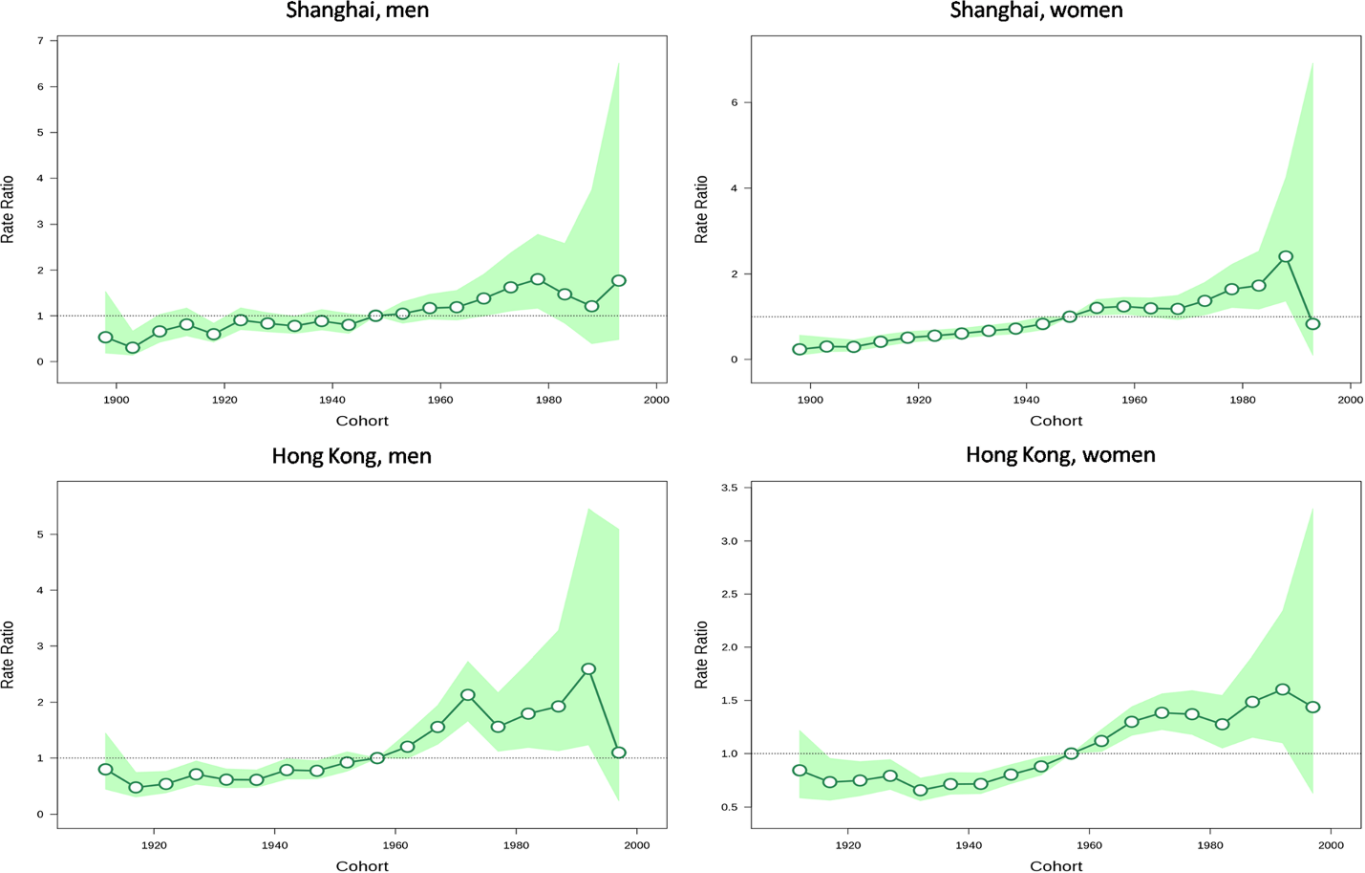
**Cohort effects on thyroid cancer incidence rates.** Cohort effects obtained from age-period-cohort analyses for the incidence rates of thyroid cancer and the corresponding 95% confidence intervals by sex in Shanghai and Hong Kong.

There were 3449 men and 11252 women, and 2457 men and 8862 women who were diagnosed with thyroid cancer in Shanghai during 1973–2009, and in Hong Kong during 1983–2011, respectively. The time trends of incidence rates of thyroid cancer by sex in Shanghai and Hong Kong are shown in Figure 1. The thyroid cancer incidence was female predominant in both populations, with the female to male ratio in age-standardized rate ranged from 1.9 to 4.5 in Shanghai and from 2.6 to 5.0 in Hong Kong across calendar years. The incidence rates of thyroid cancer in both men and women in Shanghai changed in similar patterns during the period 1973–2009. The thyroid cancer incidence decreased in men during the period 1976–1982 and in women during the period 1975–1983, followed by a slight but significant rise in men during the period 1982–2000 and in women during the period 1983–2002, and dramatically increased thereafter. We also observed increased, though not statistically significant, incidence rates in Shanghai men during the period 1973–1976 and in Shanghai women during the period 1973–1975. During the period 1973–2009, the overall AAPCs of the incidence rates in Shanghai men was 3.1% [95% confidence interval (CI): 1.0%, 5.1%] and it was 3.8% (95% CI: 1.9%, 5.7%) in women. The incidence rate of thyroid cancer among Hong Kong men steadily increased from 1983 to 2011 with a full-period AAPC of 2.2% (95% CI: 1.5%, 2.8%). We observed significant increases in the incidence rate in Hong Kong women during two periods of 1983–1989 and 2003–2011, whereas it remained stable during the period 1983–2003. The full-period AAPC in the incidence rate among Hong Kong women was 2.7% (1.6%, 3.8%). The APCs for specific period segments by sex in these two populations are presented in Table 1.The longitudinal age curves of thyroid cancer incidence by sex in Shanghai and Hong Kong were illustrated in Figure 2. The risk of thyroid cancer increased monotonically in men in both populations except for minor decreases at old ages (75 years and above), which might be explained by competing risk from deaths. The longitudinal age curve in women in Shanghai displays the similar overall pattern as men, though with a minor peak at the ages 45–49. The risk in Hong Kong women increased with age until peaking at ages 55–64 years and showed a decline thereafter.The local drift values, which indicate the APCs in the incidence rates during the study periods, by sex for specific age groups in the two populations are displayed in Figure 3. We observed local drift values above 0 in all age groups in both sexes in Shanghai, which were higher at ages 20–39 years and above 70 years in men and increased with age with a minor peak around the age of 50 years in women. The local drift values varied greatly across age groups in both sexes in Hong Kong, although most were above 0 only with a few insignificant exceptions at the oldest or youngest age groups. We observed dramatically elevated local drift values at ages 30–44 years in men and 35–44 years in women in the Hong Kong population.The estimated period and cohort effects by sex in the two populations are displayed in Figures 4 and 5, respectively. We observed period effects in similar patterns for both sexes in Shanghai, which shifted downwards since the period of 1973–1977 and then turned upwards since early 1990s with a significantly elevated risk for the periods after early 2000s in men and the periods after mid-1990s in women. The period effects remained relatively stable in both sexes in Hong Kong but yielded significantly elevated increases for the most recent period 2007–2011. The risk of thyroid cancer increased, in general, with birth cohort in both sexes in Shanghai. The cohort effects remained stable for the first several birth cohorts, followed by upwards inflection for men and women born after mid-1940s.

**Table 1:** **Time trends of age-standardized incidence rates**

Population	Calendar period	APC (95% CI)
Shanghai, men	1973-1976	17.1 (−0.6, 38.0)
	1976-1982	−16.7 (−22.6, −10.3)*
	1982-2000	2.9 (1.8, 4.1)*
	2000-2009	14.0 (10.7, 17.5)*
	***Full period***	***AAPC (95% CI) = 3.1 (1.0, 5.1)*** *
Shanghai, women	1973-1975	16.9 (−10.8, 53.1)
	1975-1983	−13.2 (−16.3, −10.0)*
	1983-2002	5.3 (4.4, 6.2)*
	2002-2009	18.4 (14.2, 22.7)*
	***Full period***	***AAPC (95% CI) = 3.8 (1.9, 5.7)*** *
Hong Kong, men	1983-2011	2.2 (1.5, 2.8)*
	***Full period***	***AAPC (95% CI) = 2.2 (1.5, 2.8)*** *
Hong Kong, women	1983-1989	6.8 (2.6, 11.2)*
	1989-2003	−0.1 (−1.0, 0.9)
	2003-2011	4.6 (2.9, 6.4)*
	***Full period***	***AAPC (95% CI) = 2.7 (1.6, 3.8)*** *

*Significantly different from zero at α = 0.05; AAPC: Average Annual Percent Change; APC: Annual Percent Change; CI: Confidence Interval.

Time trends in age-standardized incidence rates of thyroid cancer by sex in Shanghai during 1973–2009 and in Hong Kong during 1983–2011.

Wald tests suggested statistically significant cohort and period effects for both populations (P < 0.05 for all). The local drifts were statistically significant for both sexes in Hong Kong (P < 0.05 for both) but not significant in Shanghai (P = 0.376 for men, and P = 0.126 for women).

## Discussion

A worldwide upwards trend in the incidence rate of thyroid cancer has been documented in recent decades. In this study, we have described continuous increase in the incidence rate of thyroid cancer since early 1980s in two Chinese populations, in Shanghai and Hong Kong. We also observed relatively higher incidence rate during the period of early 1970s to early 1980s in both sexes in Shanghai. The increased incidence rate across populations in certain periods may be, at least partially, explained by the use of sensitive diagnostic procedures, such as the introduction of ultrasound examination in the 1970s in China and more sensitive imaging techniques (e.g. CT, MRI and PET scan) in recent years. Such speculations were also supported by the observed period effects in the age-period-cohort analyses. The increasing incidence rate would be more likely to be explained by period effects in Shanghai, given the similar changes across age groups as indicated by non-significant local drift values. On the other hand, the increasing thyroid cancer incidence may also suggest a true increase in the risk of thyroid cancer in the populations, as the magnitude of changes over time varied across age groups and the estimated cohort effects increased for recent birth cohorts.

The etiology of thyroid cancer has not been completely understood till now, whereas ionizing radiation exposure is the only unequivocally established environmental risk factor for thyroid cancer [17, 18]. A continuous increase in the frequency of medical diagnostic and therapeutic nuclear medicine procedures in Shanghai has been described during the 12 years before 2008, and the annual individual radiation dose due to medical procedures had doubled [19]. A survey by Hong Kong Census and Statistic Department in 2008 found that around 17% of the population aged 15 years and above had medical checkup regularly, among which 68% had medical checkups taken once every 7 to 12 months and one third of the checkups included X-rays [20]. More frequent use of nuclear medical procedures may have resulted in increased exposures to radiation, and thus, have contributed to the increased incidence of thyroid cancer.

Our study confirmed the female predominance in the incidence rate of thyroid cancer and also observed a downward inflection in the age curve of thyroid cancer risk after menopausal ages in women, which suggest a pivotal role of oestrogen in the development of thyroid cancer [21]. Oestrogen as a risk factor for thyroid cancer was also supported by experimental studies showing that oestrogen was a potent simulator of both human benign and malignant thyroid cells [22, 23]. If such hypothesis is confirmed with further evidence, the increasing incidence of thyroid cancer would be possible to be explained, to some extent, by the exposure to endocrine disrupting chemicals (EDCs) in the population [24, 25].

Epidemiological evidence concerning iodine intake and thyroid cancer risk remains largely lacking and controversial. Previous case–control studies reported statistically non-significant increased thyroid cancer risk associated with higher dietary intake of iodine [26, 27], while inverse associations between dietary iodine intake and thyroid cancer risk were observed in others [28, 29]. China introduced universal salt iodization in 1995 to reduce the prevalence of iodine deficiency in Mainland China [30], while no iodine supplementation program was implemented in Hong Kong. We observed a sharp increasing in thyroid cancer incidence in Shanghai since the early 2000s, around 5–7 years after the implementation of the universal salt iodination, but there was no commensurate marked increase in cohort effects in the age-period-cohort analysis. Thus, our results did not reveal a clear association between the salt iodination program and thyroid cancer risk in the population in Shanghai, which still needs to be clarified in analytic epidemiological studies.

Previous studies have indicated a higher risk of thyroid cancer in individuals with a higher socioeconomic status [31, 32]. Such observations may be explained by the increased medical radiation exposure and diet with more energy intake, which may in turn, lead to elevated body mass and consequently increased risk of thyroid cancer [33]. Our findings suggested those born after mid-1945s had increased risk of thyroid cancer, which might be linked to the improved living conditions and nutrition in the populations after the World War II. The increasing incidence rates of thyroid cancer may also be attributable to the increasing prevalence of other potential risk factors, particularly exposures to environmental carcinogens, in the populations during recent decades. The candidate exposures may include, but not restricted to, EDCs, nitrite and nitrate ingested through drinking water, solvents, and metals [18, 34]. Nevertheless, confirmation of the possible causal relationships between environmental pollutants and thyroid cancer still warrants more epidemiological investigations in the future.

Since different histological types of thyroid cancer may not be necessarily etiologically homogenous, the changing epidemiology of thyroid cancer may have varied across histological types due to the possible distinct temporal trends in the prevalence of specific risk factors in the populations. Reports from other populations have indicated that the increasing incidence rate of thyroid cancer was mostly in that of papillary thyroid cancer [35, 36]. However, we were not able to perform sub-group analyses on the time trends in the incidence rate of thyroid cancer due to lack of information on histological types, which would be a major limitation of this study. In addition, although we attempted to sort out the effects of birth cohorts and calendar periods on the observed trends in the incidence rates of thyroid cancer after adjustment for age, all etiological interpretations need to be understood with caution because of the inherent limitations of age-period -cohort analysis, such as collinearity among age, period and cohort effects. Furthermore, all time trends analyses on disease rates in this study were ecological descriptive analyses at population levels without inference at individual levels. This study was inevitably subject to ecological fallacy, since interpretations from results at population levels do not necessarily hold for individuals. Therefore, all hypotheses raised in this study still need further confirmation in analytic epidemiological studies.

## Conclusions

In summary, we observed continuous increases in the incidence rates of thyroid cancer in two Chinese populations, in Shanghai and Hong Kong, since the 1980s, in addition to higher incidence rates in the 1970s in both sexes in Shanghai. The increased incidence rates of thyroid cancer in these two Chinese populations during recent decades may be contributable to a combination of the introduction of more sensitive diagnostic techniques and the increasing prevalence of environmental exposures in the populations. More analytic epidemiological studies are warranted to clarify the underlying reasons for the increasing incidence rate of thyroid cancer and the causes of thyroid cancer.
